# The usual suspects: How psychological motives and thinking styles predict the endorsement of well‐known and COVID‐19 conspiracy beliefs

**DOI:** 10.1002/acp.3844

**Published:** 2021-05-26

**Authors:** Vukašin Gligorić, Margarida Moreira da Silva, Selin Eker, Nieke van Hoek, Ella Nieuwenhuijzen, Uljana Popova, Golnar Zeighami

**Affiliations:** ^1^ Department of Psychology University of Amsterdam Amsterdam The Netherlands

**Keywords:** conspiracy mentality, conspiracy theories, conspiratorial beliefs, COVID‐19, psychological motives, spirituality

## Abstract

Research on belief in conspiracy theories identified many predictors but often failed to investigate them together. In the present study, we tested how the most important predictors of beliefs in conspiracy theories explain endorsing COVID‐19 and non‐COVID‐19 conspiracy theories and conspiracy mentality. Apart from these three measures of conspiratorial thinking, participants (*N* = 354) completed several measures of epistemic, existential, and social psychological motives, as well as cognitive processing variables. While many predictors had significant correlations, only three consistently explained conspiratorial beliefs when included in one model: higher spirituality (specifically eco‐awareness factor), higher narcissism, and lower analytical thinking. Compared to the other two conspiratorial measures, predictors less explained belief in COVID‐19 conspiracy theories, but this depended on items' content. We conclude that the same predictors apply to belief in both COVID and non‐COVID conspiracies and identify New Age spirituality as an important contributor to such beliefs.

## 1 INTRODUCTION

How did the COVID‐19 virus turn into a worldwide pandemic? For a significant part of the population, the explanation that considers the virus to originate from a food market in China seems to be too unlikely. In the United States of America, between 15% and 28% of the population believes in an alternative explanation for the rise and spread of the COVID‐19 virus to be true (Romer & Jamieson, 2020). Such alternative beliefs—conspiracy theories—challenge the official explanations, suggesting that an event (e.g., COVID‐19 pandemic) is a malevolent act caused by a powerful group (Douglas et al., 2017). It is no surprise that conspiracy theories have gained popularity in the past year: they tend to prosper in times of crisis when people feel threatened, uncertain, and insecure (e.g., Douglas, 2021; van Prooijen & Douglas, 2017). Notably, the popularity of conspiracy theories is not only related to lower mental health (Chen et al., 2020) but also to less engagement in health‐preventive behavior during the COVID‐19 pandemic, such as distancing (Bierwiaczonek et al., 2020; Imhoff & Lamberty, 2020). Therefore, it is essential to understand what predicts belief in COVID‐19 conspiracies. In the present study, we explored how important psychological motives and cognitive factors jointly contribute to belief in COVID‐19 and non‐COVID‐19 conspiracy theories, and conspiratorial mentality.

### 1.1 Conspiracies and psychological motives

Why do people believe in conspiracy theories? Previous research suggests that people may be drawn to conspiratorial beliefs as they seem to satisfy important psychological motives. This framework identified many separate psychological motives in the literature, indicating that heightened epistemic, existential, and social motives could draw individuals to conspiracy endorsement (Douglas, 2021; Douglas et al., 2017). While the psychological motives approach provides an appropriate theoretical explanation for the relation of conspiratorial beliefs with *single* motives (or needs), it has also opened a possibility of investigating how *multiple* motives predict endorsement of conspiracy theories. The framework identifies three groups of motives as crucial in drawing individuals to conspiracy beliefs.

*Epistemic Motives*. One of the most apparent reasons to endorse conspiracy theories is one's motivation to understand and explain the world around us. Given how strong these epistemic motives are, it is no wonder that people seek meaning everywhere, even in randomness (e.g., Bar‐Hillel & Wagenaar, 1991; Zhao et al., 2014). This tendency to find patterns in random events, named *Illusory Pattern Perception* (IPP), was also found to drive conspiracy beliefs (van Prooijen et al., 2018; Whitson & Galinsky, 2008). Therefore, in an attempt to find meaning in the environment, one might end up making unfounded connections of various sorts, including endorsing conspiracies. Another important epistemic motive is the *Need for Cognitive Closure*, which consists of the urge to obtain definitive knowledge and eliminate any arbitrariness and confusion (Webster & Kruglanski, 1997). Given that conspiracy theories provide simple answers, research showed that individuals with a high need for closure and/or intolerance of uncertainty are more likely to endorse conspiracies (Farias & Pilati, 2021; Marchlewska et al., 2018; van Prooijen & Jostmann, 2013). In our study, we included both IPP and the need for cognitive closure as representative of epistemic motives.

*Existential Motives*, which are reflected in the desire for control and security, are also essential to consider as people strive to feel secure in their environments (Douglas et al., 2017). Arguably the most central such need is the *Need for Control*: according to the Compensatory Control Theory, people experience negative feelings when they lack control (Kay et al., 2009). To restore the sense of *personal* control, individuals grant more control to the *external* agents, making an equilibrium between the internal and external feelings of control. Thus, lacking individual control leads to turning to external authorities, such as religion or government. In a similar vein, conspiracy theories (which also include external agents) should compensate for lack of personal control (Kay et al., 2009). Additionally, conspiracy theories provide explanations in which events are controllable, rather than being a result of randomness, which also might strengthen the relationship between personal control and endorsing conspiracy theories. Indeed, many studies showed that people who lost control or have a high need for it endorse conspiracy theories (van Prooijen & Acker, 2015). This was shown to be the case in uncontrollable life events, such as unemployment or financial struggles (Imhoff, 2015) or the ongoing COVID pandemic (Šrol et al., 2021). However, there have been mixed results with a recent meta‐analysis showing weak effects (Stojanov & Halberstadt, 2020), so further investigation is needed. In any case, we included the need for control as a representative of existential motives.

Finally, somewhat less obvious, *Social Motives* might also play a role in conspiratorial thinking (Douglas, 2021). These motives include desires to maintain a positive view of the self or ingroup (compared to others), and conspiracies might help people achieve this. One such motive is the *Need for Uniqueness* which concerns the need to stand out from others, be anti‐conformist and experience a sense of independence (Snyder & Fromkin, 1977). Research has found that a higher need for uniqueness is related to endorsing conspiracies (Imhoff & Lamberty, 2017; Lantian et al., 2017). Hence, when people want to stand out as different, they can achieve this by endorsing uncommon views like conspiracy theories. Indeed, conspiracy theories were found to be more appealing when they were supported only by a minority (Imhoff & Lamberty, 2017). Similarly, previous studies showed that *Narcissism*, that is, grandiose perception of oneself, predicted higher belief in conspiracy theories (Cichocka, Marchlewska, & De Zavala, 2016). Therefore, one's view of herself is important in predicting conspiratorial beliefs, which is why we included both the need for uniqueness and narcissism. Since this view of oneself is always *in relation* to other people (e.g., how unique, special or grandiose one is compared to other individuals), these motives are inherently social.

#### 1.1.1 Spirituality

While previous studies focused on one of the motives, many studies focused on broader worldviews (e.g., political or religious views, Imhoff, 2015; Jasinskaja‐Lahti & Jetten, 2019). However, psychological studies on conspiratorial beliefs investigating the role of spirituality are rare. This is a large gap given that spirituality may satisfy many motives as it offers to fulfill the need for meaning or purpose in one's life (Forouzi et al., 2017), yet is distinct from religiosity (Paloutzian & Park, 2005; Willard & Norenzayan, 2017). Spirituality, like religion, has been consistently seen as a way to seek meaning in life, feel secure and connected with others (Delgado, 2005; Moxey et al., 2011), thus simultaneously fulfilling epistemic, existential, and social motives. Another reason why spirituality might have an important role in conspiratorial thinking comes from ethnology and sociology: as Ward and Voas (2011) noted, a new philosophy named “Conspirituality” has emerged, based on the core convictions of New Age spiritual beliefs and conspiracy theories. Despite some differences, there are fundamental similarities between the two, including the idea that nothing happens accidentally, or is as it seems, and that everything is connected (Barkun, 2003; Ward & Voas, 2011).

Previous studies have not thoroughly explored the relationship between conspiracy beliefs and spirituality. For example, Newheiser et al. (2011) showed that the more strongly someone endorsed New Age beliefs, the more likely they believed the Da Vinci Code conspiracy. Similarly, Marques et al. (2021) found a positive relationship between a one‐item measure that combined religion and spirituality (i.e., religion/spirituality) and belief in local and international conspiracy theories. However, both these studies had limited operationalizations of either conspiracy beliefs or spirituality—while the former investigated relation with one specific conspiracy theory, the latter combined religion and spirituality in one item.

### 1.2 Conspiracies and cognitive factors

Another fruitful line of research investigated conspiracy theories from the point of reasoning failures and/or biases. In line with the dual‐process theory of cognition (Evans & Stanovich, 2013), there are dispositions for analytical and intuitive reasoning style. While analytical style entails rule‐based and slow responses correlated with cognitive ability, intuitive is automatic and entails relying on heuristics and gut feelings (Epstein et al., 1996; Pacini & Epstein, 1999). *Analytical Thinking* has been consistently shown to be associated with lower conspiracy beliefs (e.g., Gligorić et al., 2018; Swami et al., 2014), including COVID‐19 related conspiracies (Alper et al., 2020; Stanley et al., 2020). On the other hand, given the intuitive appeal of conspiracy theories (simple but grandiose explanations), *Intuitive Thinking* is related to stronger endorsement of conspiracy theories (e.g., Denovan et al., 2020; Gligorić et al., 2018; Pytlik et al., 2020). For these reasons, we included both analytic and intuitive thinking styles in our study.

Finally, an open mind could have an important but complex role in accepting the conspiracy beliefs. First, openness as a personality trait could bring unusual, imaginative, or even paranoid ideas, which might facilitate the endorsement of conspiracy beliefs. On the other hand, given its positive relation with intelligence and cognitive styles (e.g., DeYoung et al., 2012), it might serve as a protective factor against epistemically suspect beliefs by employing a critical mind (Bainbridge et al., 2019). Indeed, studies have shown support for both relations, corroborating both positive (e.g., Swami et al., 2011, 2013) and negative associations (Rizeq et al., 2020; Swami et al., 2016) between openness and conspiracy beliefs. For this reason, we included three facets of openness from the HEXACO model, one focusing on unusual ideas (*Unconventionality*), one on knowledge‐seeking (*Inquisitiveness*), and the last on artistic creativity (*Creativity*) (Ashton & Lee, 2007).

### 1.3 The aims of the present study

Research reviewed above brought many insights about individual correlates of conspiracies. However, there is still little empirical groundwork in understanding how these variables work together and which ones are more relevant. Only rarely have studies made strides to assess the joint contribution of various predictors (e.g., Marques et al., 2021). This is problematic, especially because many psychological motives or concepts overlap (Douglas et al., 2017), so exploring individual correlates might fail to assess the relative importance of predictors. In the present study, we aimed to contribute to the little work that has been put forward about the joint contribution of previously identified variables to belief in conspiracies and to group them into two main predictor groups: psychological motives and cognitive factors.

Secondly, we wanted to explore the role of spirituality in conspiratorial beliefs because earlier research has only measured this relation using either spirituality related measurements (Jasinskaja‐Lahti & Jetten, 2019), conspiracy related constructs (Willard & Norenzayan, 2017), or specific conspiracy theories (Newheiser et al., 2011). This gap is surprising given that Douglas et al. (2017; Douglas, 2021) suggest that conspiratorial beliefs could fulfill psychological motives, with spirituality being an important such need (van Dierendonck, 2012).

Finally, our third aim was to test whether reviewed individual correlates predict the COVID‐19 related conspiracy theories in the same way as they predict other specific conspiracy theories and conspiracy mentality. In this way, we tested the generalizability of formerly discovered predictors. Although the research on belief in COVID‐19 conspiracies is on the rise (van Mulukom et al., 2020), many previous studies focused on single predictors of belief in these conspiracies (e.g., analytical thinking, Swami & Barron, 2020; uncertainty, Farias & Pilati, 2021; control, Oleksy et al., 2021; Šrol et al., 2021). We wanted to test these predictors' relative importance, therefore informing future research about which predictors to focus on more. This is an important aim given that belief in conspiracy theories negatively impacts health behavior such as handwashing, distancing (Imhoff & Lamberty, 2020), or vaccination (Hornsey et al., 2018).

## 2 METHOD

### 2.1 Participants

The minimum number of participants we set on was 238, based on the sample size needed for correlations of .2 to stabilize (80% critical point of stability). However, to achieve more power, we aimed to approach the number of 362 participants, based on the sample size needed for correlations of .1 to stabilize (90% critical point of stability) (Schönbrodt & Perugini, 2013). Through convenience sampling and snowballing, we recruited 402 participants who completed the survey voluntarily. After excluding the participants who failed one of the attention checks (39) and multivariate outliers based on Mahalanobis distance (9), the final sample size was 354. This sample (35.3% male, 63.0% female, and 1.7% other) was made up of participants ranging between 16 and 68 years, with a mean age of 28.6^1^ (SD = 11.3). Participants identified as mostly white (78.2%), but also Asian/Pacific Islander (8.2%), Hispanic/Latino (2.5%), Black/African American (2.3%), or indicated “Other” ethnicity (8.8%). Finally, the sample was highly educated, with most of the participants having a degree (29.1% had an undergraduate, while 29.7% had a graduate degree) or studying (32.2%). The rest had a high school degree (7.6%) or a degree lower than high school (1.4%).

### 2.2 Procedure

Participants filled out an online survey programmed in Qualtrics that was distributed via social media by six undergraduate students (all of the European background) at the University of Amsterdam. The survey was part of a larger joined project (approved by the Ethical Review Board of the University of Amsterdam) and also included a scale of pseudo‐profound bullshit. However, given that it was part of a different research aim, we do not mention it in this paper. Before filling out the questionnaire, participants were administered an information brochure, which informed the participant of the study's goal and procedure, guaranteed privacy, and voluntary participation. The participants confirmed their consent by clicking on the designated button. The survey took around 20 minutes to complete.

### 2.3 Materials

The complete questionnaire with all items and sources is given at the Open Science Framework (OSF; osf.io/nc9jz). Two attention checks were embedded in the survey (“This is an attention check question. Please answer ‘Strongly agree’”) within the scales measuring the Need for control and Narcissism.

#### 2.3.1 Conspiratorial beliefs

We included three measures of conspiratorial beliefs. We measured *Belief in Specific Conspiracy Theories* (CTs) by selecting five common CTs (e.g., “The moon landing is a hoax” and “The HIV/AIDS virus has been genetically engineered to wipe out certain sectors of the population”) to which participants indicated their agreement (1 = “definitely not true” to 5 = “definitely true”) (van Prooijen et al., 2018). The scale showed good reliability (*α* = .78).

To estimate the *Belief in COVID‐19 CTs*, the participants indicated their agreement with three items (e.g., “I believe the coronavirus was created in a laboratory according to plans unknown to the public” and “I believe there are groups interested in spreading panic to achieve their own goals”) using a five‐point scale (1 = “strongly disagree” to 5 = “strongly agree”) (Oleksy et al., 2021; study 1). This short scale showed satisfactory reliability (*α* = .66).

Finally, to measure *Conspiracy Mentality* as a more general conspiratorial mindset, we used the Conspiracy Mentality Scale (Stojanov & Halberstadt, 2019). The scale consists of seven items, such as: “The government or covert organizations are responsible for events that are unusual or unexplained”. Participants responded to the items on a seven‐point Likert scale (1 = “strongly disagree” to 7 = “strongly agree”). The scale had high reliability (*α* = .88).

#### 2.3.2 Psychological motives

*Illusory Pattern Perception* (IPP). Participants rated the extent to which they saw a pattern in five “chaotic” modern paintings with higher ratings indicating higher IPP. We selected five (out of nine) paintings that van Prooijen et al. (2018) used, and participants answered to what extent they saw a pattern in each painting (1 = “not at all” to “very much”). Two filler questions about the painting concerning beauty and familiarity were included to conceal the measure's goal. The scale had good reliability (*α* = .82).

*Need for Cognitive Closure*. To assess the need for cognitive closure, we used the 15 item Need for Closure Scale (NFC; Roets & van Hiel, 2011). Participants rated statements such as “I feel uncomfortable when I don't understand the reason why an event occurred in my life” using a five‐point Likert scale ranging from 1 = “strongly disagree”, to 5 = “strongly agree”. The scale showed high reliability (*α* = .82).

*Need for Control* was measured using the factor of General Desire for Control from the Desirability of Control Scale (DCS; Burger & Cooper, 1979). The scale consisted of six items (e.g., “I try to avoid situations where someone else tells me what to do”) to which participants responded using a seven‐point Likert scale (1 = “strongly disagree”, to 5 = “strongly agree”). The scale showed high reliability (*α* = .82).

*Need for Uniqueness*. To measure the need for uniqueness, we used the Self‐Attributed Need for Uniqueness Scale (SANU; Lynn & Harris, 1997). Participants rated four statements (e.g., “I prefer being different from other people”) on a five‐point Likert scale ranging from 1 = “strongly disagree”, to 5 = “strongly agree”. This scale showed high reliability (*α* = .86).

*Narcissism* was measured using Narcissistic Admiration and Rivalry Questionnaire Short Scale (NARQ‐S; Leckelt et al., 2018). Participants rated six items (e.g., “I react annoyed if another person steals the show from me”) using a six‐point answering scale (1 = “strongly disagree”, to 6 = “strongly agree”). This scale showed satisfactory reliability (*α* = .72).

*Spirituality* was measured with the 23‐items Spirituality Scale (SS; Delaney, 2005). The scale is composed of three factors: *Self‐discovery* (four items, e.g., “I have a sense of purpose”; *α* = .75), *Relatedness* (six items, e.g., “I value maintaining and nurturing my relationships with others”; *α* = .60), and *Eco‐Awareness* (13 items, e.g., “I have a relationship with a Higher Power/Universal Intelligence” and “I meditate to gain access to my inner spirit”; *α* = .91). Participants indicated their agreement with the statements on a five‐point Likert scale ranging from 1 = “strongly disagree” to 5 = “strongly agree”. Scores were calculated both for each subscale and the whole scale. The entire scale showed high reliability (*α* = .90).

#### 2.3.3 Cognitive processing

*Analytical Cognitive Style*. To measure preference for employing an analytical cognitive style, participants completed the 5‐item Need for Cognition scale of the 10‐item Rational‐Experiential Inventory (REI‐10; Epstein et al., 1996). Participants rated items (e.g., “I prefer complex to simple problems”) using a five‐point scale (1 = “completely false” to 5 = completely true”). The scale had satisfactory reliability (*α* = .72).

*Intuitive Cognitive Style*. We selected five items with the highest loadings from the Experiential Engagement subscale of the 40‐item Rational‐Experiential Inventory (REI‐40; Pacini & Epstein, 1999), measuring participant's preference for employing an intuitive thinking style. Participants rated statements (e.g., “I like to rely on my intuitive impressions”) on a five‐point scale (1 = “completely false” to 5 = “completely true”). The scale showed good reliability (*α* = .76).

Finally, we selected three facets of the personality trait of Openness to Experience. We measured *Unconventionality* as a maladaptive variant of openness with four items (e.g., “I think of myself as a somewhat eccentric person”; *α* = .50) from the 100‐item HEXACO inventory (Lee & Ashton, 2018). *Inquisitiveness*, which measures knowledge‐seeking, was assessed using two items (e.g., “I'm interested in learning about the history and politics of other countries”; *r* = .32), while *Creativity* was measured with three items (e.g., “I would enjoy creating a work of art, such as a novel, a song, or a painting”; *α* = .72), both taken from the 60‐item version of the HEXACO (Ashton & Lee, 2009). Items were rated from 1 = “strongly disagree” to 5 = “strongly agree”.

## 3 RESULTS

Data and the analysis script can be found at the OSF. Means, standard deviations, and intercorrelations of variables are given in Table 1. Several correlation patterns are noteworthy. First, all three measures of belief in CTs correlated highly with each other indicating the constructs' similarity. These high positive correlations are in line with previous studies which argue that different conspiracy beliefs form a monological belief system. Similar to the conspiracy measures, Spirituality subcomponents (Self‐Discovery, Relatedness, Eco‐Awareness) had medium to high intercorrelations. However, these subcomponents correlated differently with belief in CTs—Self‐Discovery had a low positive correlation while Relatedness did not correlate at all. On the other hand, Eco‐Awareness had medium‐sized positive correlations (*r*s > .38), suggesting that the relationship between Spirituality and conspiratorial beliefs is due to this factor. Regarding the epistemic needs, the Need for Cognitive Closure and IPP positively correlated with two out of three measures of conspiracy theories (relationship with the belief in COVID‐19 CTs was not significant). Need for Control positively correlated with all three measures of conspiratorial belief. Investigating the social motives, Need for Uniqueness did not correlate with any of conspiratorial measures, while Narcissism positively correlated with all of them. Regarding cognitive processing variables, Analytical thinking showed a negative relationship with all measures of belief in CTs. On the other hand, Intuitive thinking style was positively related only to Conspiracy Mentality. Investigating the openness personality trait showed that Unconventionality was positively related to Conspiracy Mentality, while the factor of Inquisitiveness was negatively related to Belief in specific CTs and Conspiracy Mentality. Finally, Creativity was unrelated to conspiratorial beliefs.

**TABLE 1:** Means, standard deviations, and correlations

Variable	*M* (SD)	1	2	3	4	5	6	7	8	9	10	11	12	13	14	15	16
1. Belief in specific CTs	2.00 (0.80)																
2. Belief in COVID CTs	2.99 (0.94)	.63^**^															
3. Conspiracy mentality	3.44 (1.29)	.68^**^	.67^**^														
4. IPP	3.21 (1.38)	.12^*^	.10	.15^**^													
5. Need for closure	3.16 (0.62)	.16^**^	.08	.14^**^	−.25^**^												
6. Need for control	4.71 (1.09)	.14^**^	.14^**^	.15^**^	.11^*^	−.01											
7. Need for uniqueness	3.34 (0.95)	.05	.09	.10	.17^**^	−.08	.38^**^										
8. Narcissism	3.01 (1.00)	.28^**^	.21^**^	.26^**^	.15^**^	.21^**^	.39^**^	.40^**^									
9. Self‐discovery	3.81 (0.81)	.15^**^	.16^**^	.07	.21^**^	−.01	.23^**^	.23^**^	.11^*^								
10. Relatedness	4.40 (0.44)	.04	.02	.03	.12^*^	.06	.08	.16^**^	−.01	.50^**^							
11. Eco‐awareness	2.93 (0.92)	.38^**^	.38^**^	.40^**^	.21^**^	.12^*^	.12^*^	.21^**^	.17^**^	.46^**^	.32^**^						
12. Spirituality	3.47 (0.65)	.34^**^	.34^**^	.34^**^	.24^**^	.10	.16^**^	.24^**^	.16^**^	.67^**^	.54^**^	.95^**^					
13. Analytical thinking	3.72 (0.73)	−.23^**^	−.14^**^	−.20^**^	.11^*^	−.41^**^	.22^**^	.17^**^	−.09	.23^**^	.18^**^	−.07	.03				
14. Intuitive thinking	3.50 (0.73)	.09	.05	.14^**^	.20^**^	−.13^*^	.15^**^	.20^**^	−.01	.18^**^	.21^**^	.16^**^	.21^**^	.11^*^			
15. O. Unconventionality	3.62 (0.67)	.04	.07	.11^*^	.18^**^	−.38^**^	.18^**^	.36^**^	.10	.03	.04	.08	.07	.31^**^	.15^**^		
16. O. Inquisitiveness	3.82 (0.98)	−.24^**^	−.08	−.15^**^	.08	−.22^**^	.05	.10^*^	−.06	.05	.05	.00	.02	.34^**^	−.07	.28^**^	
17. O. Creativity	3.71 (0.97)	−.02	.02	.04	.23^**^	−.22^**^	.10	.20^**^	.07	.13^*^	.24^**^	.18^**^	.21^**^	.20^**^	.10	.34^**^	.20^**^

*Note: M* and SD are used to represent mean and standard deviation, respectively. ^*^ *p* < .05. ^**^ *p* < .01.

Abbreviations: IPP, illusory pattern perception; O, openness.

It is important to note that several predictors (IPP, Need for Closure, Narcissism, Analytical and Intuitive thinking styles, and Inquisitiveness factor of Openness) showed lower correlations with belief in COVID‐19 CTs than with the two other conspiracy measures. Although these differences in correlation might not necessarily be significant, they indicate that predictors might explain less variance of Belief in COVID‐19 conspiracies than of the other two conspiracy measures.

We next tested how psychological motives and cognitive factors predicted conspiracy beliefs by conducting three linear regression analyses for each conspiracy measure. As Table 2 shows, Spirituality was the most important predictor (highest coefficient) for all three conspiracy measures, indicating that higher scores in Spirituality are related to higher belief in conspiracies. Likewise, lower Analytical thinking style and higher Narcissism were associated with all measures of conspiratorial beliefs. Interestingly, two factors of Openness (Unconventionality and Inquisitiveness) were associated with belief in specific CTs and conspiracy mentality, but not with Belief in COVID‐19 CTs. Finally, the Need for Uniqueness was negatively associated with the belief in specific CTs. However, given their low positive zero‐order correlation, the association in the model is likely a suppressor effect, thus not worth interpreting. Looking at the overall model, predictors explained less variance for COVID‐19 CTs (*R* ^2^ = .18) than specific CTs (*R* ^2^ = .28) or Conspiracy Mentality (*R* ^2^ = .25). However, prediction of separate COVID‐19 conspiracy theories showed large variability: the proportion of explained variance for conspiracies regarding the *benefits of some groups* (“I believe there are groups interested in spreading panic to achieve their own goals” and “I believe that the development of the pandemic may benefit certain groups of whose interests we have no idea”) was lower (*R* ^2^s = .08 and .11 respectively) than it was about the *virus origin* (“I believe the coronavirus was created in a laboratory according to plans unknown to the public”; *R* ^2^ = .25). Therefore, it is important to take content into account when predicting the beliefs in conspiracy theories.

**TABLE 2:** Three regression models with usual suspects predicting three measures of conspiracy beliefs

Model (DV and fit)	Belief in specific CTs *F*(11,342) = 12.15, *p* < .001; *R* ^2^ = .28 (adjusted *R* ^2^ = .26)			Belief in COVID‐19 CTs *F*(11,342) = 6.82, *p* < .001; *R* ^2^ = .18 (adjusted *R* ^2^ = .15)			Conspiracy mentality *F*(11,342) = 10.26, *p* < .001; *R* ^2^ = .25 (adjusted *R* ^2^ = .22)		
Predictor	*β*	*t*	*p*	*β*	*t*	*p*	*β*	*t*	*p*
IPP	.05	1.05	.30	.01	.22	.82	.07	1.41	.16
Need for closure	.01	.22	.83	−.04	−.68	.50	.05	.90	.37
Need for control	.08	1.49	.14	.09	1.56	.12	.08	1.43	.16
Need for uniqueness	**−.14**	**−2.50**	**.01**	−.07	−1.22	.22	−.10	−1.77	.08
Narcissism	**.21**	**3.70**	**<.001**	**.13**	**2.11**	**.04**	**.16**	**2.72**	**<.01**
Spirituality	**.33**	**6.46**	**<.001**	**.34**	**6.30**	**<.001**	**.30**	**5.71**	**<.001**
Analytical thinking	**−.19**	**−3.37**	**<.001**	**−.17**	**−2.91**	**<.01**	**−.20**	**−3.60**	**<.001**
Intuitive thinking	.02	.42	.68	−.02	−.33	.74	.07	1.28	.20
O. Unconventionality	**.15**	**2.77**	**< .01**	.11	1.89	.06	**.20**	**3.56**	**<.001**
O. Inquisitiveness	**−.19**	**−3.83**	**<.001**	−.05	−.95	.34	**−.12**	**−2.21**	**.03**
O. Creativity	−.07	−1.29	.20	−.06	−1.09	.28	−.04	−.76	.45

*Note*: Significant predictors are in bold.

Abbreviations: IPP, illusory pattern perception; O, openness.

Finally, we wanted to test which factor of Spirituality was the most important predictor by including three subcomponents of Spirituality (Self‐Discovery, Relatedness, Eco‐Awareness) in the same regression models we ran previously (while leaving out Spirituality). In predicting the Belief in specific CTs, only Eco‐Awareness emerged as a significant predictor (*β* = .33, *t* = 6.01, *p* < .001), but not Self‐Discovery nor Relatedness (*β*s < .04, *p*s > .58). The same pattern emerged for predicting Belief in COVID‐19 CTs (*β* = .36, *t* = 6.16, *p* < .001) and Conspiracy Mentality (*β* = .39, *t* = 6.99, *p* < .001), with neither Self‐Discover or Relatedness attaining significance (*β*s < .10, *p*s > .12). This supports the notion from the intercorrelation table that the positive relationship between Spirituality and conspiracy beliefs is due to the Eco‐Awareness component. Multicollinearity was not an issue in any of the regressions above, with all variance inflation factors below 1.74.

## 4 DISCUSSION

In the present study, we investigated how a large number of predictors related to psychological motives and thinking styles jointly explain belief in specific conspiracy theories (both related and unrelated to COVID‐19) and conspiratorial mentality. Although many predictors showed significant individual correlations, together only three variables consistently emerged as significant in explaining three conspiratorial measures: spirituality (driven by eco‐awareness factor), analytical thinking, and narcissism. Openness to unusual ideas (unconventionality) was associated with higher belief in specific CTs and conspiracy mentality, while openness to information and knowledge (inquisitiveness) negatively predicted these two conspiratorial measures. None of the openness measures predicted belief in COVID‐19 CTs.

Given the high intercorrelations between measures of conspiratorial belief, our study demonstrates that conspiracy theories about COVID‐19 do not differ in any special way from other conspiracy theories, supporting the idea of a monological belief system (Goertzel, 1994; Miller, 2020; Swami et al., 2011). That is, our findings support the idea that it does not matter whether conspiracies are measured as a general conspiracy mentality or as different specific conspiracy beliefs because these might tap into the same construct, though on different levels of specificity (e.g., Bruder et al., 2013; Stojanov & Halberstadt, 2019). Therefore, our study informs the debate on whether the conspiratorial belief system is monological with separate conspiracies relying on each other (Hagen, 2018). Equally important, the same predictors account for different conspiratorial beliefs, supporting the idea that COVID‐19 CTs do not have an exceptional status though the pandemic is taking place. However, it seems that the content of the CT matters (Oleksy et al., 2021) as we found a stronger association of predictors with the conspiracy theory regarding the virus origin than two conspiracy theories about the benefits of certain groups. We believe this is because the former resembles the traditional conspiracy theories more, in that it is a strong and clear statement about secret plot contradictory to official explanations, while the latter simply refers to benefiting from the given situation, but without ascribing much power and control. Future research should investigate the importance of the content more.

Our study corroborated the importance of spirituality, analytical thinking, and narcissism for conspiracy beliefs. While the finding on analytical thinking is in line with previous research (e.g., Stanley et al., 2020; Swami et al., 2014), we showed it to be a protective factor even after important psychological motives are taken into account. Therefore, future research should employ this elaborate and reflective reasoning in trying to reduce conspiratorial beliefs, as Orosz et al. (2016) did. Similarly, given that information‐seeking (inquisitiveness facet of openness) was associated with lower belief in specific CTs and conspiracy mentality, it is clear that knowledge and rationality could prove very beneficial in countering conspiracy beliefs. Interestingly, narcissism emerged as one of the most important predictors of endorsing CTs, while the need for uniqueness, though moderately correlated with narcissism (*r* = .40), did not. The absence of the relationship between conspiratorial beliefs and the need for uniqueness is not in line with previous research (Imhoff & Lamberty, 2017; Lantian et al., 2017), though it is noteworthy that such research corroborated relatively small correlations varying between .1 and .2. This also suggests that the predictive power of narcissism is less because of one's feeling of being special, but rather other factors such as paranoid thought (Cichocka, Marchlewska, & De Zavala, 2016) or possibly because both narcissism and conspiracy theories include grandiosity, so narcissist's beliefs about the world would be as grandiose as they are about herself. In any case, while past research focused more on collective narcissism (e.g., Cichocka, Marchlewska, De Zavala, & Olechowski, 2016; Marchlewska et al., 2019), future research would also benefit from investigating individual narcissism more.

### 4.1 The role of spirituality

Most surprisingly, spirituality, particularly its eco‐awareness factor, emerged as the most significant predictor of higher conspiracy endorsement. On its face value, the eco‐awareness factor mostly resembles New Age spirituality since it includes beliefs such as connections between all things, the existence of higher intelligence, and mediation. Therefore, the importance of such spirituality in predicting conspiracy beliefs is in line with findings on the positive relationship with Da Vinci conspiracy theory (Newheiser et al., 2011). This relationship is also in line with the view of Conspirituality, that is, that New Age spirituality and conspiratorial beliefs may converge to one worldview, conjoining the features of unusual (paranormal) beliefs, secret societies, and knowledge, and interconnectedness in the world (Asprem & Dyrendal, 2019; Barkun, 2003; Ward & Voas, 2011). It is likely that susceptibility to paranormal beliefs, of which New Age spirituality represents an integral part (Tobacyk, 2004), is behind this relationship, given the positive association between paranormal and conspiracy beliefs (e.g., Darwin et al., 2011; Lobato et al., 2014). Another possibility is their attraction to alternative explanations and lifestyles, which would be in line with findings that believers in the benefits of alternative medicine endorse more conspiratorial beliefs (Galliford & Furnham, 2017). In any case, future research should investigate more directly why the strong positive relationship emerges between eco‐awareness spirituality and conspiracy beliefs.

On the other hand, the other two factors of Delaney's (2005) conception of spirituality were less important for conspiratorial beliefs. Namely, the factor of self‐discovery which includes seeking and finding meaning (resembles epistemic motives) was related to belief in specific conspiracies (both related and unrelated to COVID‐19), but only as zero‐order correlations. This gives some support to the notion that conspiracy theories can have epistemic functions (Douglas, 2021). Finally, relatedness is an important part of spirituality (Delaney, 2005), and while it resembles the need for belonging (social motive), we did not find a relationship with conspiratorial beliefs. Therefore, from social motives perspective, conspiratorial beliefs seem to be driven by anti‐conformity (being special, unique, grandiose), rather than being conformist (need to belong to a group). This is not to say that group membership is irrelevant for conspiratorial beliefs (many conspiracy theories are concerned with outgroup members), but that in two competing needs of an individual (need to be different vs. need to belong; Hornsey & Jetten, 2004), conspiratorial beliefs draw on the former.

### 4.2 Limitations, contributions, and conclusion

We had several limitations in our study. First, we did not include all psychological motives (e.g., motives relating to groups). However, we did include that representative of epistemic, existential, and social motives, attempting to outline how these motives predict conspiratorial beliefs when joined together. Secondly, our measures were often too short, possibly not estimating the relationships precisely. However, given the large number of constructs we included, it would be hardly possible to use longer scales. Additionally, most of the measures showed good reliability, and the stable relationships were detected despite the scales' shortness (e.g., the five‐item measure of analytic thinking), indicating that the construct is more important than the length of scale used. Finally, one of the limitations is the sampling method as we used convenience sampling and snowballing to collect the responses through social media, which could have led to a biased sample. However, given that six students who recruited participants come from different European countries, it is very likely that the sample was diverse (as evidenced in the sample structure), increasing the external validity of the results. Additionally, meta‐psychological research showed that heterogeneity of psychological effects is more contingent on the effect itself (i.e., phenomenon) than the sample used (Klein et al., 2018). While this limitation is therefore attenuated, future research should test the generalizability of our findings on different samples.

On the other hand, our research made several important contributions. We tested a large number of predictors to estimate their relative importance, an approach rarely undertaken in the literature. Next, our study corroborated spirituality, and particularly eco‐awareness, which resembles New Age spirituality, as a potentially very fruitful construct in explaining conspiratorial beliefs. Finally, we compared COVID‐19 and other conspiracy theories, showing that they are similar, forming a monological belief system. To conclude, different conspiracy theories are psychologically very similar, and New Age spirituality might play an important role in such beliefs.

## CONFLICT OF INTEREST

Authors have no conflict of interest.

## ETHICS STATEMENT

The Ethical approval was granted by the Institutional Review Board of the Department of Psychology at the University of Amsterdam.

## Data Availability

All data and code are available in the links provided in the main text

## Endnote

^1^
Due to a mistake in survey programming, we did not record age for 55 participants, so the given mean applies to 299 participants
